# Control of Bacillus subtilis Replication Initiation during Physiological Transitions and Perturbations

**DOI:** 10.1128/mBio.02205-19

**Published:** 2019-12-17

**Authors:** John T. Sauls, Sarah E. Cox, Quynh Do, Victoria Castillo, Zulfar Ghulam-Jelani, Suckjoon Jun

**Affiliations:** a Department of Physics, University of California, San Diego, La Jolla, California, USA; b Section of Molecular Biology, Division of Biology, University of California, San Diego, La Jolla, California, USA; University of Utah

**Keywords:** cell cycle, cell size, replication initiation, single cell

## Abstract

Bacillus subtilis and Escherichia coli are evolutionarily divergent model organisms whose analysis has enabled elucidation of fundamental differences between Gram-positive and Gram-negative bacteria, respectively. Despite their differences in cell cycle control at the molecular level, the two organisms follow the same phenomenological principle, known as the adder principle, for cell size homeostasis. We thus asked to what extent B. subtilis and E. coli share common physiological principles in coordinating growth and the cell cycle. We measured physiological parameters of B. subtilis under various steady-state growth conditions with and without translation inhibition at both the population and single-cell levels. These experiments revealed core physiological principles shared between B. subtilis and E. coli. Specifically, both organisms maintain an invariant cell size per replication origin at initiation, under all steady-state conditions, and even during nutrient shifts at the single-cell level. Furthermore, the two organisms also inherit the same “hierarchy” of physiological parameters. On the basis of these findings, we suggest that the basic principles of coordination between growth and the cell cycle in bacteria may have been established early in evolutionary history.

## Abstract (precis)

High-throughput, quantitative approaches have enabled the discovery of fundamental principles describing bacterial physiology. These principles provide a foundation for predicting the behavior of biological systems, a widely held aspiration. However, these approaches are often exclusively applied to the best-known model organism, E. coli. In this report, we investigate to what extent quantitative principles discovered in Gram-negative E. coli are applicable to Gram-positive B. subtilis. We found that these two extremely divergent bacterial species employ deeply similar strategies in order to coordinate growth, cell size, and the cell cycle. These similarities mean that the quantitative physiological principles described here can likely provide a beachhead for others who wish to understand additional, less-studied prokaryotes.

## INTRODUCTION

Our current understanding of fundamental, quantitative principles in bacterial physiology is largely based on studies of Escherichia coli and Salmonella enterica serovar Typhimurium (see a previous report by Jun et al. [1] for a review of the history and recent progress). Several of these principles have been presented in the form of “growth laws” (1). For example, the growth law of cell size states that the average cell size increases exponentially with the nutrient-imposed growth rate (λ) (2). This principle has been extended to the growth law of unit cells, which allows prediction of the average cell size based on the growth rate and the cell cycle duration under any steady-state growth condition (3).

Gram-positive Bacillus subtilis is distinct from Gram-negative E. coli at the genetic, molecular, and regulatory levels (4). However, despite their evolutionary divergence, B. subtilis and E. coli follow the same phenomenological principle of cell size homeostasis known as the adder principle (5, 6). Furthermore, the two organisms share identical mechanistic origins of the adder principle, namely, a molecular threshold for division proteins and their balanced biosynthesis during growth (7). On the basis of these findings, we wanted to know to what extent B. subtilis and E. coli coordinate growth, size, and cell cycle in the same manner. A shared coordination framework would imply that, despite phylogenetic and molecular diversity, physiological regulation in bacteria is functionally conserved.

In order to create a full complement of the data necessary for comparative analysis, we measured the growth and cell cycle parameters of B. subtilis at both the population and single-cell levels under a wide range of conditions.

Previous population-level studies found that B. subtilis, like E. coli, initiates replication at a fixed mass, establishing a regulatory bridge between cell size and cell cycle control (8–10). We extended this avenue with single-cell methods to precisely measure the cell cycle parameters in individual B. subtilis cells across conditions (7, 11). These results showed that the initiation size per *ori* (*s_i_*) is constant under steady-state conditions as well as during nutrient shifts between two steady-state conditions. This strongly supports a threshold model for initiation in both static and dynamic environments (3, 7, 12, 13).

The single-cell approach also allowed us to compare the relative levels of variability of all growth and cell cycle parameters both between conditions and between species. These measurements reveal strikingly similar hierarchies of physiological parameters between B. subtilis and E. coli in terms of the tightness of their control.

The richness of our quantitative physiological data generated in B. subtilis is comparable to that in E. coli, providing key evidence that B. subtilis and E. coli share core phenomenological and quantitative principles that govern their physiology. These principles provide a unified picture of bacterial growth, size, and cell cycle coordination.

## RESULTS AND DISCUSSION

### Ensuring steady-state growth in B. subtilis.

Maintaining steady-state growth is essential for reproducible measurements of the physiological state of the cell (1). In steady-state growth, the total biomass of the culture increases exponentially with time and protein biosynthesis is balanced with the total biomass increase. That is, the protein production rate is the same as the growth rate of the cell. As a result, average protein concentrations are constant, whereas the total amount of proteins increases in proportion to the cell volume. The constant concentration and the proportional increase also apply to other macromolecules such as DNA, RNA, phospholipids, and the cell wall.

**FIG 1:**
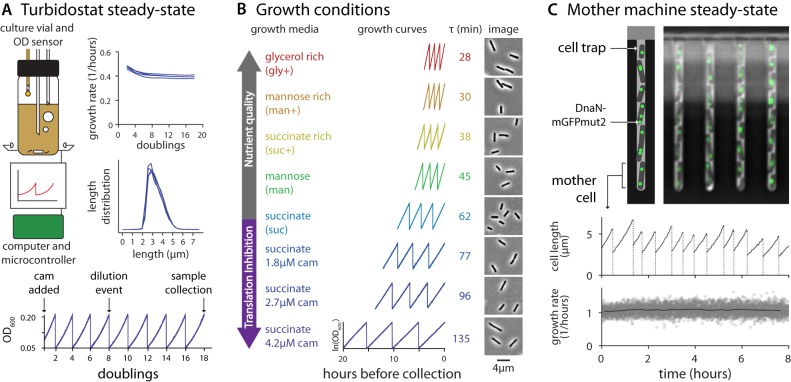
Population and single-cell methods to achieve steady-state growth. (A) Turbidostat experimental method and validation. (Top left) In the multiplex turbidostat vial, the culture volume was maintained at a constant level and the cell concentration was monitored and adjusted automatically by infusing fresh medium. Aerobic conditions were ensured via bubbling and stirring. (Top right) Growth rate measurements were consistent between 5 and 20 doublings, and cell length distributions were reproducible at sample collection. Data shown are from 4 repeats in succinate with 2.7 μM chloramphenicol (cam). (Bottom) A representative growth curve showing the timing of the addition of chloramphenicol, dilution events, and sample collection. Each dilution occurred when the culture reached an OD_600_ of 0.2 and was diluted to 0.05, allowing for two doublings during the growth interval. (B) Overview of population growth conditions and measurements. Growth media and their abbreviations represent 5 different nutrient conditions, all of which are based on S7_50_. Glycerol-rich, mannose, and succinate media were selected for translation inhibition experiments (succinate results are shown here). Representative growth curves (final 8 doublings), average doubling time (τ), and representative crops of images used for population sizing are shown for each condition. (C) Single-cell experiments performed with the mother machine. A representative image showing cell-containing traps is shown. The fluorescent signal represents DnaN-mGFPmut2 (see Fig. 3 and Materials and Methods). The growth in length (black lines) and division (dotted vertical lines) of a single mother cell is shown over 8 h. Average growth rate data (solid gray lines) from the single-cell measurements (gray scatter points) represent steady-state conditions over the course of the experiment. Data shown are from mannose conditions. Data from additional measurements performed under all conditions are presented in Fig. S1.

To achieve steady-state measurements in B. subtilis, we grew and monitored cells over many generations using a multiplex turbidostat that we had previously used for E. coli (3) (Fig. 1A). For both the population and single-cell assays, we began cultures from single colonies and precultured cells using appropriate batch methods before transferring them to continuous culture setups (see Materials and Methods). To avoid sporulation, we ensured that the precultures did not enter stationary phase. We used a B. subtilis strain which was nonmotile and nonbiofilm forming to facilitate size measurements of single cells. This was necessary because B. subtilis exhibits a temporal chaining phenotype, particularly under conditions mediating faster growth (14, 15). During chaining, cells are physically connected and yet their cytoplasms are compartmentalized, obfuscating a definition of division (16, 17). Our strain contained a genetic modification to abolish cell chaining, ensuring that cell separation coincided with septation (18) (Materials and Methods).

10.1128/mBio.02205-19.1 FIG S1 Single-cell steady-state physiological parameters under all conditions. Physiological parameters are shown for all B. subtilis mother machine experimental conditions and one E. coli experiment. Time course is shown with single-cell measurements (scatter points) and 30-minute binned mean (horizontal lines) plotted against the birth time. Multiple consecutive generations are needed to determine initiation size, C period, and D period; thus, a time gap exists before those measurements are possible. Single-cell distributions are invariant in time and shown for each condition, sharing the same scale as the time course data. Colors are as described for Fig. 1B. Sample sizes are provided in Table 2. Download FIG S1, PDF file, 12.6 MB. Copyright © 2019 Sauls et al. 2019 Sauls et al. This content is distributed under the terms of the Creative Commons Attribution 4.0 International license.

To measure how long B. subtilis takes to reach physiological steady state, we measured growth rate continuously during time course experiments using our multiplex turbidostat. Growth rate generally stabilized after 6 generations, and the cell size distribution was reproducible (Fig. 1A). However, to be certain of steady-state growth, we typically waited for at least a total of 14 doublings before sample collection was performed in all our subsequent experiments. At collection, we split the culture for quantitative PCR (qPCR) marker frequency analysis and cell size measurement (see Table 1 for experimental conditions).

**TABLE 1:** Turbidostat experimental conditions for strain BS15

Growth medium	Perturbation	No. of replicates	No. of cells per experiment
succinate	None	4	6,592, 8,769, 7,418, 7,051
succinate	1.8 μM cam^*a*^	4	16,804, 11,418, 15,001, 8,065
succinate	2.7 μM cam	4	7,051, 7,901, 13,369, 7,741
succinate	4.2 μM cam	2	4,782, 3,132
mannose	None	2	4,782, 3,132
mannose	1 μM cam	3	5,368, 9,213, 8,086
mannose	2 μM cam	4	14,080, 12,865, 18,524, 15,191
mannose	3.5 μM cam	3	6,623, 7,218, 9,140
succinate rich	None	2	7,861, 18,963
mannose rich	None	2	3,346, 3,387
glycerol rich	None	3	4,807, 3,152, 4,819
glycerol rich	1 μM cam	3	3,551, 4,894, 2,199
glycerol rich	2 μM cam	4	7,821, 3,332, 9,554, 7,973
glycerol rich	3.5 μM cam	4	5,786, 5,143, 7,059, 4,910

a cam, chloramphenicol.

For single-cell measurements, we used the microfluidic mother machine to collect phase-contrast and fluorescent time-lapse images for at least 10 generations (7, 19) (Fig. 1C). After analyzing durations of all cell lives, we limited our data to the time interval in which all measured parameters equilibrated (see Fig. S1 in the supplemental material). A typical experiment produced data for around 2,500 cells (see Table 2 for experimental conditions). We used custom software to extract single-cell data from raw images (20) (Materials and Methods).

**TABLE 2:** Mother machine experimental conditions

Strain	Growth medium	Perturbation	Total no. of cells	No. of cells with initiation size
BS45	Succinate	None	2,530	506
BS45	Succinate	2 μM cam	2,586	534
BS45	Mannose	None	5,478	504
BS45	Mannose	2 μM cam	3,375	561
BS45	Mannose	3.5 μM cam	2,151	553
BS45	Glycerol rich	None	2,355	514
BS45	Glycerol rich	2 μM cam	4,743	476
BS45	Glycerol rich	3.5 μM cam	1,416	198
BS43	Succinate, succinate rich	Nutrient shift	7,671	1695
SJ1724	MOPS glucose	None	4,681	437

### Growth law of cell size: B. subtilis size shows a positive but not exponential dependence on the nutrient-imposed growth rate.

A foundational observation by Schaechter, Maaløe, and Kjeldgaard showed that the average cell size in E. coli increases exponentially with the nutrient-imposed growth rate (2). Previously, we investigated this “growth law of cell size” in E. coli under various growth and cell cycle inhibition conditions and showed that the exponential relationship was a special case wherein the growth rate was the only experimental variable (3). In B. subtilis, the Levin laboratory recently revisited the relationship between size and the nutrient-imposed growth rate and found that the average cell size in B. subtilis increased with the growth rate at the population level (21).

We extended our efforts in E. coli to B. subtilis. Using the multiplex turbidostat, we grew cells under 5 nutrient conditions with doubling times ranging between 28 and 62 min (Fig. 1B; see also Materials and Methods and Table 1). Here, we used size interchangeably with volume and considered volume to be proportional to dry mass (22).

**FIG 2:**
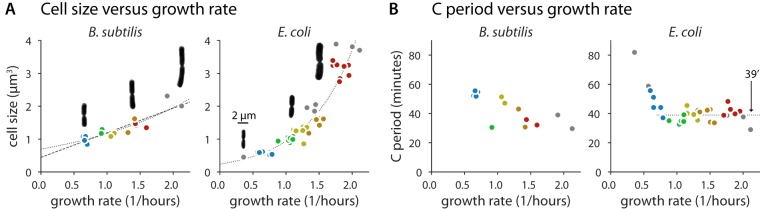
Population cell size and C period measurements in B. subtilis and E. coli. (A) Cell size increases with respect to growth rate in B. subtilis and E. coli under conditions of nutrient limitation. The relationship is not as clearly exponential for B. subtilis as it is for E. coli (dotted lines are linear regression fits of logarithm-transformed data, the dashed line is a linear regression fit). Representative images of cells during division show change in aspect ratio as a function of growth rate. Length and width measurements are presented in Fig. S2. (B) C period measurements with respect to growth rate in B. subtilis and E. coli under conditions of nutrient limitation. For E. coli, C is approximately constant at 39 min (horizontal dotted line) for doubling times faster than 60 min (λ = 0.69). The two-sided *P* value to reject the null hypothesis that the slope of C on growth rate is zero is 0.55 using the Wald test. The constancy is less clear for B. subtilis, with a *P* value of 0.13 for the same test. Though lower, it is still not enough to reject the null hypothesis that the slope is zero. However, single-cell data show that C + D is proportional to generation time (Fig. S5). B. subtilis growth media are colored as described for Fig. 1B, with additional LB data in gray. The E. coli data are from previously published work (3). Red, synthetic rich; orange, glucose with 12 amino acids; yellow; glucose with 6 amino acids; green, glucose; blue, glycerol. Additional conditions are indicated in gray.

Panel A of Fig. 2 shows the average cell size versus growth rate under the 5 different growth conditions. As expected, the average cell size increased with growth rate. However, the exponential dependence observed for E. coli was less clear in B. subtilis. This difference in B. subtilis could have been due to changes in the durations of replication (C period) and cell division (D period) under different nutrient conditions (3).

10.1128/mBio.02205-19.2 FIG S2 Length and width measurements in B. subtilis and E. coli. (A) Cell length in B. subtilis and E. coli increases with growth rate. (B) For B. subtilis, width is independent of the nutrient-imposed growth rate. For E. coli, width increases with growth rate in a manner similar to the length. Colors and conditions are as described for Fig. 2. E. coli data are from previously published work (3). Download FIG S2, PDF file, 0.1 MB. Copyright © 2019 Sauls et al. 2019 Sauls et al. This content is distributed under the terms of the Creative Commons Attribution 4.0 International license.

10.1128/mBio.02205-19.5 FIG S5 B. subtilis cell cycle and initiation size behavior. (A) The value corresponding to the C period plus the D period (C + D) is proportional to generation time in B. subtilis when the generation time is modulated by nutrient condition or translational inhibition. (B) The cell width seen under conditions of translational inhibition decreases compared to the average width in the absence of inhibition (*W*_0μm_) (dotted black line). (C) Initiation length increase with width under conditions of translational inhibition. Mean initiation length per *ori* (l*i)* shown as a dotted black line. (D) Initiation size from Fig. 4A reproduced for comparison. In all plots, colors are as described for Fig. 4, where lines connect the same growth media with and without chloramphenicol. Scatter points represent single-cell data, and solid symbols represent population averages. Download FIG S5, PDF file, 0.8 MB. Copyright © 2019 Sauls et al. 2019 Sauls et al. This content is distributed under the terms of the Creative Commons Attribution 4.0 International license.

We thus measured the population average C period of B. subtilis employing qPCR marker frequency analysis (3, 9, 23). The two species exhibited similar maximum replication speeds (approximately 40 min for the C period). However, our data for B. subtilis are too sparse to determine if or for which growth rates the C period was constant (Fig. 2B).

Unfortunately, despite extensive efforts, we were unable to reliably measure the D period in B. subtilis from the population samples as we had done previously for E. coli (3). The main issue was a lack of consistency of fluorescence labeling of the DNA required for flow or image cytometry. Our results were variable from experiment to experiment and from protocol to protocol. We therefore concluded that the measurement of D period using population methods was not as reliable as needed to test the growth law of cell size in B. subtilis, a cautionary reminder in interpreting previous measurements in B. subtilis. For these reasons, we set out to measure the B. subtilis cell cycle explicitly at the single-cell level.

### Single-cell determination of cell cycle parameters in B. subtilis.

We employed a functional replisome protein fused with a fluorescent marker, DnaN-mGFPmut2, to measure cell cycle progression in single cells (7, 24) (Materials and Methods). In B. subtilis, the replisomes from the two replication forks of a replicating chromosome are often colocalized; thus, most foci represent a pair of replisomes (25).

**FIG 3:**
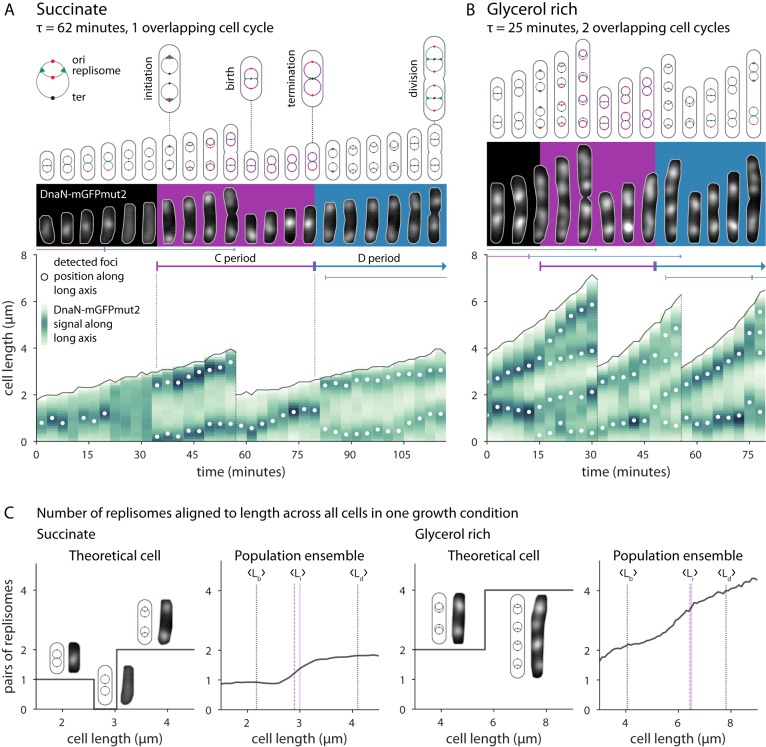
Single-cell growth and cell cycle progression in B. subtilis. (A) Typical cell cycle progression of B. subtilis in media mediating slower growth. All panels show the same two cells. (Top) Chromosome configuration and key cell events. (Middle) Fluorescent images of DnaN-mGFPmut signal. Gray outlines are from the segmented phase-contrast image. Purple and blue backgrounds indicate C and D periods corresponding to the second division. (Bottom) Processed image data represented as a cell lineage trace. Cell length and division are indicated by the solid and dotted black lines, respectively. Vertical green bars represent the DnaN-mGFPmut2 signal summed along the long axis of the cell, with white circles showing focus positions. The single-cell initiation size, C period, and D period are determined manually from these traces. (B) Typical cell cycle progression of B. subtilis in media mediating faster growth. (C) Ensemble method to determine cell cycle parameters. (Left) In succinate, a theoretical cell is born with one pair of replisomes. It may briefly contain no active replisomes upon termination and then contain two pairs of replisomes as the two complete chromosomes begin replication. The length to which the numbers of replisome pairs increases corresponds to the initiation size. Across all cells, the average number of replisome pairs transitions from one to two at the population’s initiation size. The average initiation length (L_i_) as determined from the cell traces (dashed purple line) agrees with the ensemble estimate (solid purple line). The average birth length (L_b_) and division length (L_d_) of the population are shown as dotted vertical lines. (Right) In glycerol-rich media, cells transition from two to four pairs of replisomes. Images of ensembles under all conditions are shown in Fig. S4.

Panels A and B of Fig. 3 show representative cells under two growth conditions, succinate and glycerol rich, respectively. Under the condition mediating slower growth (succinate), cells were normally generated with one replicating chromosome. Replication initiation begins synchronously in the mother cell for two chromosomes. At that time, the origins are located toward the cell poles. Replication proceeds through cell division, at which point the replication forks reside near the midcell of the newly generated cell. Chromosome segregation is concurrent with replication. By the time the replication forks reach the terminus region, which is still at the midcell, the previously duplicated origins have already migrated to the cell poles (26).

10.1128/mBio.02205-19.3 FIG S3 Size and C period under conditions of translational inhibition in B. subtilis and E. coli. (A) Under conditions of translation inhibition due to the presence of chloramphenicol, the relationship between cell size and growth rate under conditions of nutrient limitation breaks down for both B. subtilis and E. coli. (B) The deviation from the nutrient growth law can be attributed to the change in the C period in both species under conditions of translation inhibition. Lines connect data from translation inhibition experiments performed using the same media. Colors and conditions are as described for Fig. 2. E. coli data are from previously published work (3). Download FIG S3, PDF file, 0.2 MB. Copyright © 2019 Sauls et al. 2019 Sauls et al. This content is distributed under the terms of the Creative Commons Attribution 4.0 International license.

10.1128/mBio.02205-19.4 FIG S4 Ensemble replisome count and localization under all conditions. Ensemble plots for three sets of medium conditions tested with and without translational inhibition. Data representing averages of results from pairs of replisomes (thick black line) are plotted against cell volume with a consistent scale across conditions. As in Fig. 3C, purple vertical lines show the initiation size from the average results determined using single cells (dashed) and the ensemble method (solid). Vertical dotted black lines indicate the average birth and division sizes. We can calculate the average number of foci for sizes outside the average birth and division length due to cell-to-cell variability (ensemble data are shown at sizes to which at least 50 cells contributed). The average number of foci may be above or below the theoretical number. This is because replisomes transiently dissociate and because a pair of replisomes may be counted as two foci when they are not colocalized (25, 61, 62). The normalized DnaN-mGFPmut2 signal relative to the signal seen midcell (green background) shows the localization of replisomes over the cell cycle, with the diagonal solid black lines indicating the cell periphery. Termination and replication initiation are often synchronous and correspond to bifurcations in the localization pattern. Download FIG S4, PDF file, 0.6 MB. Copyright © 2019 Sauls et al. 2019 Sauls et al. This content is distributed under the terms of the Creative Commons Attribution 4.0 International license.

It is common for initiation to have occurred in the previous generation even under conditions of slow growth (i.e., the total of the combined C and D periods [C + D] is greater than the doubling time [τ]). Yet cells rarely exhibit multifork replication. In multifork replication, initiation begins before the previous termination event completes, such that a single replicating chromosome possesses four or even eight copies of *ori*. Instead, B. subtilis normally initiates when the cell contains complete, homologous chromosomes where the copy number represents a power of 2. In fact, replication initiation often proceeds immediately after the previous termination event. This may be due to the role of YabA in B. subtilis replication initiation control, which ties DnaA activity to DnaN availability (27, 28). Multifork replication is comparatively common in E. coli, where Hda is thought to play a similar but mechanistically distinct role in reducing initiation potential during ongoing replication (7, 29).

Under conditions mediating faster growth (glycerol-rich conditions), cells are large and often born with two replicating chromosomes. These large cells then simultaneously initiate replication at four *ori*. However, the relative levels of variability between division size (*S_d_*) and C + D were greater under this rich condition. This means that a substantial fraction of the population was smaller and born with one replicating chromosome and consequently initiated at two *ori* (Fig. S8). Moreover, transient filamentation and asymmetrical septation are more common under conditions mediating fast growth, leading to the generation of cells born with numbers of replicating chromosomes which do not represent a power of 2.

### Complementary ensemble determination of cell cycle parameters in B. subtilis.

The main advantage of the single-cell approach is that it allows direct comparisons of the relationships between growth parameters, providing mechanistic insights (6). However, it can be difficult to determine the cell cycle parameters directly, particularly when the foci are clumped or the signal is weak. This is especially true under conditions mediating faster growth. To ensure an unbiased analysis of the cell cycle, we also employed an ensemble method, compiling data from many individual cells and extracting the average cell cycle parameters (11) (Fig. 3C). We used the focus count at a given size as a proxy for the replication state (Materials and Methods). This method produces data similar to those generated by the original schematics used by Cooper and Helmstetter when they first elucidated the E. coli cell cycle (30).

Under all conditions except those mediating the slowest growth, the measured average number of foci monotonically increases because initiation almost immediately follows termination, as discussed above. Unlike data corresponding to a theoretical single cell, the ensemble plots do not display a strict step-like behavior; we interpret this as variability in the initiation size. Ensemble plots determined under all conditions, along with the focus localization patterns, are presented in Fig. S4. The data are in good agreement with the average initiation size as measured from individual cells. We used these complementary methods to test whether the initiation size in B. subtilis is as invariant as in E. coli (3).

### Invariance of initiation size: B. subtilis initiates at a fixed cell size per *ori*.

The concept of a conserved initiation size in E. coli and S. enterica serovar Typhimurium was first explained by Donachie as a consequence of the growth law of cell size and the constant C + D (2, 8, 30). The upshot is that, at a fixed size per origin (*ori*), all origins of replication fire simultaneously. Recent high-throughput work performed at both the single-cell and population levels (3, 7, 11) conclusively showed that the early insight reported by Donachie was correct. In fact, the initiation size per *ori* is invariant not only across nutrient conditions but also under conditions of antibiotic inhibition and genetic perturbations (3).

**FIG 4:**
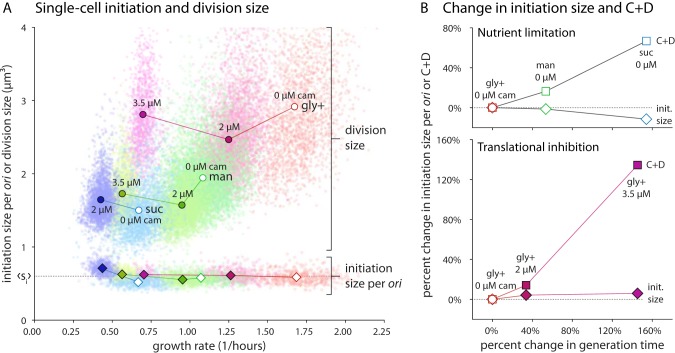
Initiation size is invariant in B. subtilis during steady-state growth. (A) Single-cell initiation size per *ori* s_i_ is condition independent. Division size (circles) changes dramatically under both nutrient limitation and translational inhibition conditions. The corresponding initiation size per *ori* (diamonds) collapses onto a constant value (s_i_) across all conditions (dotted horizontal black line). This holds for both single cells (scatter points) and population averages (solid symbols). Growth media are colored as described for Fig. 1B, with the amount of chloramphenicol indicated (0 μM chloramphenicol with empty symbols; lines connect the same growth media with and without chloramphenicol). (B) C + D values are condition dependent and increase with generation time. (Top) Increases in generation time under conditions of nutrient limitation cause an increase in the population average C + D (squares), while initiation size changes only minimally. (Bottom) A similar pattern is seen under conditions of translational inhibition. For both plots, measured parameters are compared to the condition mediating fastest growth. Single-cell C + D data presented in Fig. S5 and Fig. S8.

The constancy of initiation size in B. subtilis at the population level under nutrient limitation conditions was previously tested by several groups (9, 10, 31). In nutrient limitation, the initiation size was found to be constant, though it can be decreased in some mutants. We measured the initiation size using single-cell methods under nutrient limitation and translational inhibition conditions (Table 2). We found that the initiation size per *ori* in B. subtilis is indeed invariant across conditions, even for individual cells (Fig. 4A).

This constant initiation size is in stark contrast to the varying C period seen under different growth conditions (Fig. S5A). In fact, initiation size is one of the least variable physiological parameters along with septum position and width (Fig. S8). The single-cell approach also allowed us to measure the correlations between all growth and cell cycle parameters. The initiation size was found to correlate only weakly with other measured parameters (Fig. S9).

10.1128/mBio.02205-19.8 FIG S8 Unnormalized physiological parameter distributions under all conditions. Data represent B. subtilis physiological parameter distributions under all conditions. Mean (μ) and CV data are presented in the legends. The true initiation size (*Si*) is the size at initiation not corrected for the number of *ori* genes (bottom middle panel). Under glycerol-rich conditions, cells may be born with 1 or 2 replicating chromosomes. This results in a bimodal distribution of the true initiation size depending on whether they have two or four *ori* genes at the time of initiation, as the initiation size per *ori* is constant in either case. Initiation size per *ori s_i_*, width, and septum position are the most highly conserved parameters across growth conditions. Download FIG S8, PDF file, 0.3 MB. Copyright © 2019 Sauls et al. 2019 Sauls et al. This content is distributed under the terms of the Creative Commons Attribution 4.0 International license.

10.1128/mBio.02205-19.9 FIG S9 Normalized cross-correlations. Data represent normalized cross-correlations under all B. subtilis conditions and one E. coli condition. Lines represent linear regression fits to the single-cell data. Symbols are as described for Fig. 6. Normalized distributions are plotted along the diagonal. Download FIG S9, PDF file, 0.9 MB. Copyright © 2019 Sauls et al. 2019 Sauls et al. This content is distributed under the terms of the Creative Commons Attribution 4.0 International license.

These observations are consistent with a threshold model for replication initiation (3, 7, 32). Within that framework, initiator molecules accumulate in a manner proportional to the growth rate. This mechanism is employed in single cells and is in turn apparent at the population level.

### Initiation size is invariant at the single-cell level even during nutrient shifts.

Because individual cells had shown a constant initiation size in the previous steady-state experiments, we wondered how cells would behave in a changing environment. Nutrient shift experiments have provided important insight into the coordination of biosynthesis and the cell cycle (33–35). We revisited this paradigm at the single-cell level, shifting cells from minimal media (time [τ] = 65 min) to rich conditions (τ = 30 min) and back again (Fig. S6). By using the mother machine, we could add and remove nutrients immediately while measuring the cell cycle and all other physiological parameters (Materials and Methods).

**FIG 5:**
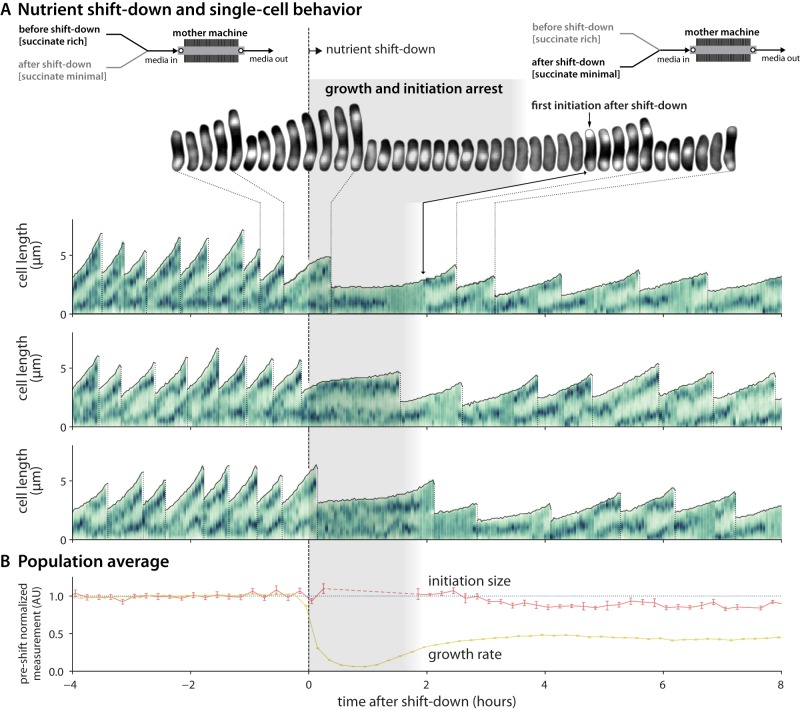
Initiation size is invariant in B. subtilis during the shift down. (A) Behavior of single cells undergoing the shift down from succinate-rich to succinate-minimal conditions at time zero. Upon the shift down, cells pause growth and initiation for 1 to 2 h. The medium shift is achieved via a Y valve upstream of the mother machine inlet. (Top) Fluorescent images of cell lineage during the shift down. (Bottom) Representative traces of 3 lineages. Representation is as described for Fig. 3, with the density of the vertical green bars representing the intensity of the DnaN-mGFPmut2 signal along the long axis of the cell. (B) Population average behavior during the shift down. Growth rate data represent the instantaneous elongation rate (6-min time step). Initiation size per *ori* is plotted against the initiation time. Each measurement is normalized to the corresponding mean in the 4 h before the shift down. Lines connect the 12-min binned mean, and error bars represent standard errors of the means. The minimum bin size is 5. The dashed-line portion of the line representing average initiation size signifies a gap in initiation events after the shift down. *n* = 3,160 cells (752 with initiation size). The entire time course showing the shift up and the shift down is available in Fig. S6.

The most drastic results occurred upon the shift down (Fig. 5). When nutrient supplements were removed, growth immediately paused. The crash in the growth rate caused a drastic increase in generation and cell cycle times for the cells which experienced the shift down. Replicating chromosomes were stalled, and division ceased (although division did occur immediately after the shift for the replication cycles already in D period). Strikingly, the growth pause led to an absence of initiation events until after cells restarted elongation and attained the requisite initiation size. Thus, individual cells maintained a constant initiation size through the transition.

Division also resumed after growth recommenced, but at a smaller cell size commensurate with the growth rate seen after the shift down. During the shift down, and despite the growth pause, the division rate per unit growth increases, while the initiation rate per unit growth stays constant. This means that the *ori*/cell ratio, which is high under conditions of fast growth, decreases until a new steady-state level is reached. The duration of the C + D periods is also not constant during this time (Fig. S6).

10.1128/mBio.02205-19.6 FIG S6 Initiation size is invariant during the shift up and the shift down. (A) Representative lineage trace for cells undergoing the shift up at time zero followed by the shift down 12 h later. Note that this particular trace exhibits transient filamentation before and unrelated to the shift down. (B) Population average behavior for all cells. Mean lines are calculated as described for Fig. 5, except that the measurements are normalized by the respective means seen in the 4 h before the shift up. Additionally, birth size is plotted against the birth time and C + D data are plotted against the corresponding division time. Upon the shift up, the growth rate immediately increased, simultaneously resulting in an increase in birth size. C + D decreased proportionally. After the shift down, all parameters return to their preupshift averages. Despite the complex dynamics of these parameters during nutrient shifts, the initiation size showed a change of less than 10% during the entire time course. *n* = 7,671 cells (1,695 with initiation size and C + D). Download FIG S6, PDF file, 5.2 MB. Copyright © 2019 Sauls et al. 2019 Sauls et al. This content is distributed under the terms of the Creative Commons Attribution 4.0 International license.

The decoupling of initiation and division supports the idea that they are controlled by independent threshold mechanisms (7). That is, the cell builds up a pool of dedicated molecules for each task to reach a certain level (7, 12, 36–38). For initiation, this threshold and the accumulation rate are conserved across growth conditions. For division, the threshold or the accumulation rate is set by the growth condition (39). In the generation after the shift down, cells grow much more slowly and therefore accumulate threshold molecules at a similarly depressed rate. As a result, both initiation and division are delayed. For division, active degradation or antagonization of FtsZ could further hinder the triggering of constriction (40, 41).

### E. coli and B. subtilis change cell shape differently under different growth conditions but maintain a constant initiation size.

One of the major differences between E. coli and B. subtilis is that their cells change shape differently under different nutrient conditions. Data from our laboratory and others have shown that the aspect ratio of E. coli is nearly constant (at a value of approximately 4) under conditions of different nutrient-imposed growth rates (3, 42). In contrast, the average width of B. subtilis remains relatively constant (Fig. S2) (10, 43).

Nevertheless, for initiation control in B. subtilis, we found that the volume per *ori* is more highly conserved than the length per *ori* at initiation. While we found length to be a good proxy for initiation size under conditions of nutrient limitation, our data show that chloramphenicol treatment decreased cell width in B. subtilis. Thus, across all growth conditions, only the initiation volume was constant (Fig. S5B to D).

### B. subtilis and E. coli share the same hierarchy of physiological parameters.

**FIG 6:**
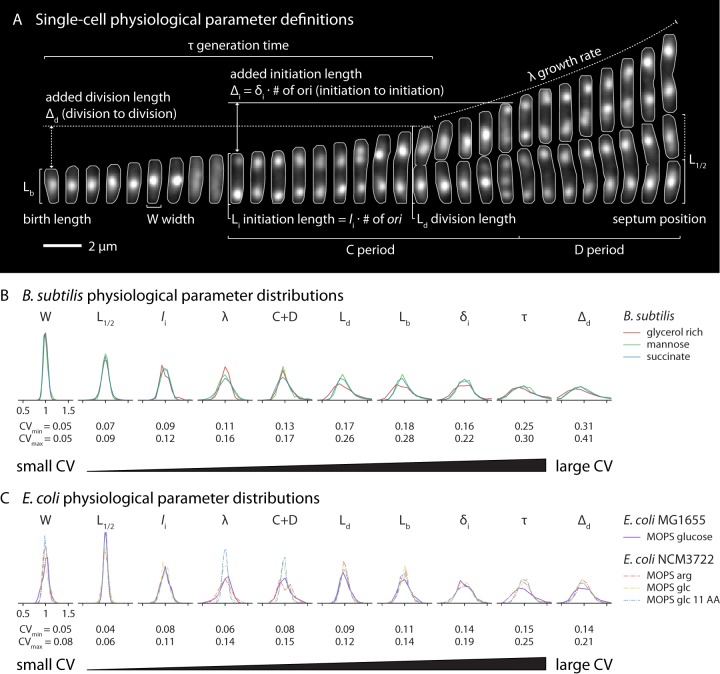
B. subtilis and E. coli share the same hierarchy of physiological parameters. (A) Single-cell physiological parameter definitions as determined from time-lapse images. The cells were B. subtilis growing in mannose (τ = 38 min). The fluorescent signal represents DnaN-mGFPmut2, and the gray outlines are from the segmented phase-contrast image. The picture interval was 3 min. (B) B. subtilis parameter distributions are shown in order of ascending coefficient of variation (CV) values. Parameters are normalized to their means. The range of CVs for each parameter is shown below the distributions. Note that length-based parameters are shown here; their volume equivalents have slightly higher CVs due to variability in width. (C) Distribution of the same measurements in E. coli displaying the same CV hierarchy. Data representing E. coli NCM3722 grown in MOPS arginine (arg), glucose (glc), and glucose plus 11 amino acids (glc 11 AA) are from previously published work (7).

The coefficient of variation (CV) of a distribution of a physiological parameter is often interpreted as the tightness of the underlying biological control (44). We extended previous analysis to include the cell cycle-related parameters C period, D period, initiation size, and added size (Δ*_d_*) at initiation for both B. subtilis and E. coli. We found that the two evolutionarily distant organisms shared the same order of their physiological parameters in terms of CV (Fig. 6). Width, septum position, initiation size, and growth rate represented the most tightly controlled parameters. The D period was significantly more variable than the C period, and they are inversely correlated. In fact, the CVs of a particular physiological parameter were found to be extremely similar across growth conditions, species, and strains (Fig. S7). Because the imaging frequency dictates the precision of our measurements, the actual CVs of the parameters are likely slightly (1% to 2%) lower than the values presented (Materials and Methods).

10.1128/mBio.02205-19.7 FIG S7 Normalized physiological parameter distributions under all conditions. (A) B. subtilis parameter distributions from perturbation experiments are commensurate with nutrient limitation conditions. The C period had a smaller CV than the D period. (B) In E. coli, the CV of the C period was smaller than that of the D period. The CV of C + D was lower than that of each individually as they are inversely related. Data from E. coli NCM3722 grown in MOPS arginine (arg), glucose (glc), and glucose plus 11 amino acids (glc 11 AA) are from previously published work (7). Download FIG S7, PDF file, 0.3 MB. Copyright © 2019 Sauls et al. 2019 Sauls et al. This content is distributed under the terms of the Creative Commons Attribution 4.0 International license.

The CVs of the physiological parameters are not all independent, and we previously showed analytically how the generation time (τ), division size (*S_d_*), birth size (*S_b_*), and added size (Δ*_d_*) are related (6). Qualitatively, this can be understood because, for example, the birth length is determined by the division length and the septum position. Therefore, the CV of birth length is greater than the CV of division length due to the contribution of the small variability in the septum position. Similarly, the CV of division size *S_d_* is larger than that of the initiation size *S_i_* because of the additional contribution from the variability of the C and D periods as well as the growth rate (λ). This can be seen most clearly for the conditions mediating slow growth with no overlapping replication cycles, where the division size is determined as *S_d_* = *S_i_* exp[(C + D)λ]. Therefore, the variabilities of C, D, λ, and *S_i_* all contribute to the variability of *S_d_*; i.e., the variability of *S_d_* must be greater than that of *S_i_*.

Ultimately, the CV of the physiological parameters represents the manifestation of molecular regulatory mechanisms. Classically, B. subtilis and E. coli provide excellent examples of both homologous and nonhomologous versions of such mechanisms. For example, major protein players controlling replication and division, such as DnaA and FtsZ, are conserved in these and most other prokaryotes (45, 46). However, the regulation of those molecules in B. subtilis and E. coli is unique (47–49). More generally, the two species often use unrelated mechanisms to achieve the same regulatory goal (49, 50). Because of their phylogenetic distance, the uncanny agreement between the CVs of their physiological parameters suggests that an evolutionarily ancient control framework is shared by these organisms.

### Summary and outlook.

**FIG 7:**
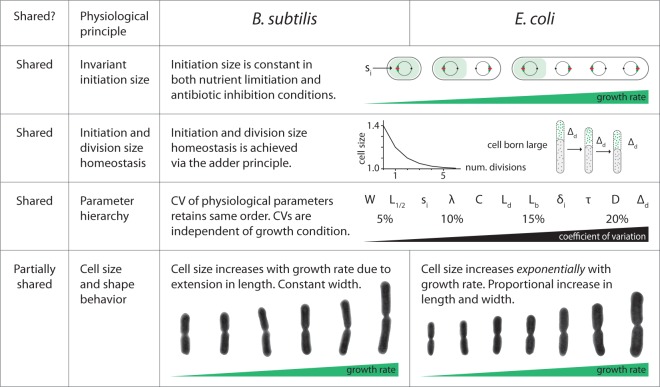
B. subtilis and E. coli comparative summary.

We have shown that B. subtilis and E. coli, despite their historical separation represented by the Gram stain divide, share extremely similar fundamental physiological behaviors (Fig. 7). Under a wide range of nutrient and growth inhibition conditions, both species base their chromosome replication on a constant initiation size. Impressively, this constant initiation size is imposed even during dynamic growth transitions.

Mechanically, our data support previous findings showing that B. subtilis is both a division adder and initiation adder (6, 7, 51) and therefore suggest that initiation is controlled in a manner similar to division. That is, the invariance of initiation mass can be explained by the following two requirements for the adder phenotype (7): (i) constant production of initiator molecules at the same rate as cell elongation (“balanced biosynthesis”) and (ii) their accumulation to a threshold number to trigger initiation. For example, as the growth rate changes upon nutrient shift, the production of the initiator molecules changes by the same rate such that the concentration remains nearly constant. As long as the threshold number of the initiator molecules is independent of the growth condition, the initiation mass must remain invariant.

As with E. coli, DnaA and FtsZ are among the key proteins responsible for the initiation and division threshold mechanisms in B. subtilis, respectively (7, 13, 52). They can be considered initiator molecules, though not necessarily to the exclusion of others with which they work in concert. The view that global biosynthesis fundamentally controls their production, and thus the replication and division rate, is also compatible with the idea that additional levels of regulation modulate or coordinate their activity in certain situations (48, 53). It is unclear whether these additional mechanisms have evolved to increase replication and division fidelity during steady-state or are more important in dynamic environments. More single-cell shift experiments performed with mutant or even minimal genome cells will help reveal the importance of redundant regulatory systems. This line of study can also experimentally address the limits on variability of physiological processes and why some cells seem to control some processes more tightly than others (54).

These deep similarities between B. subtilis and E. coli speak to a conserved control framework which both species use to coordinate growth, DNA replication, and division. In doing so, they ensure that life’s essential demand of physiological homeostasis is met. In the end, it is unclear if this framework is the result of parallel evolution or of convergent evolution. In order to better address this issue, more high-quality single-cell data are needed from diverse prokaryotes. In any case, the existence of a shared control framework underscores its efficacy, providing an intriguing avenue for the development of synthetic organisms.

## MATERIALS AND METHODS

### Strains.

We used B. subtilis strains in the 3610 background with mutations to confer nonmotility and reduce biofilm formation, a kind gift from Petra Levin (6). The background strain contained *comI*(Q12L) to confer competence (55). Dan Kearns also graciously provided an inducible *lytF* construct to enable prevention of chaining (18). For the mother machine experiments in which replisomes were tracked, we used *dnaN-mGFPmut2*, an instrumental gift from Paul Wiggins and Alan Grossman (24, 25). Strain construction was performed using single-crossover plasmid recombination or double-crossover recombination from genomic DNA (56).

For E. coli, we used a K-12 MG1655 strain containing a functional *dnaN-YPet* construct, considerately shared by Rodrigo Reyes-Lamothe (57). Strain genotypes for both species are provided in Table 3.

**TABLE 3:** Strain information

Strains	Genotype	Notes
B. subtilis strains		
BS15	*3610 comI*(Q12L) *hag*::MLS(R)::MLS(S)^*a*^ *amyE*::[Phyperspank- *lytF spcR*] *epsH*::*tet*	Gift from Petra Levin; used for turbidostat expts
BS45	*3610 comI*(Q12L) *motAB*::*Tn*917 *amyE*::[Physpank-*lytF kan*] *epsH*::*tet dnaN*::[*dnaN*-*gfp speC*]	This study; used for microfluid and turbidostat expts
E. coli strain		
SJ1724	K-12 MG1655 *dnaN*::[*dnaN*-*yPet kan*] *hupA*::[*hupA*-*mRuby2* *FRT*-*cat*-*FRT*]	This study; used for microfluid expts

a MLS(R), macrolide, lincosamide, streptogramin resistant; MLS(S), macrolide, lincosamide, streptogramin susceptible.

### Growth media and experimental conditions.

For B. subtilis, we used S7_50_ medium with different carbon sources and supplements (Sigma-Alrich, MO). Importantly, we included additional iron(III) chloride and trisodium citrate. The latter acts as a siderophore for B. subtilis, and without it our strain cannot grow in the mother machine (58). To make rich conditions, we added 2 mg/ml Casamino Acids and 0.04 mg/ml tryptophan. For E. coli, we used MOPS (morpholinepropanesulfonic acid) glucose medium. Turbidostat and mother machine experiments used the same media with the following emendation: bovine serum albumin was added at 0.5 mg/ml during the mother machine experiments in order to reduce cell adherence to surfaces inside the device. Tables 4 and 5 provide detailed information on the composition of the media.

**TABLE 4:** Growth media

Medium	Abbreviation	Carbon source	Nitrogen source	Buffer, salts, and metals	Supplement
S7_50_ succinate	suc	1% succinate	0.1% glutamate	S7_50_ salts and metals	
S7_50_ mannose	man	1% mannose	0.1% glutamate	S7_50_ salts and metals	
S7_50_ glycerol	gly	1% glycerol	0.1% glutamate	S7_50_ salts and metals	
Succinate-rich S7_50_	suc^+^	1% succinate	0.1% glutamate	S7_50_ salts and metals	0.2 mg/ml Casamino Acids, 0.04 mg/ml tryptophan
Mannose-rich S7_50_	man^+^	1% mannose	0.1% glutamate	S7_50_ salts and metals	0.2 mg/ml Casamino Acids, 0.04 mg/ml tryptophan
Glycerol-rich S7_50_	gly^+^	1% glycerol	0.1% glutamate	S7_50_ salts and metals	0.2 mg/ml Casamino Acids, 0.04 mg/ml tryptophan
MOPS Glucose	MOPS glc	0.2% glucose	9.5 mM ammonium chloride	MOPS modified buffer	
LB	LB	0.5% glucose			1% tryptone, 0.5% yeast extract, 0.05% NaCl

**TABLE 5:** Media components

Component	Concn
S7_50_ salts and metals	
MOPS	50 mM
Ammonium sulfate	1 mM
Potassium phosphate monobasic	5 mM
Magnesium chloride	2 mM
Calcium chloride	0.7 mM
Manganese(II) chloride	50 μM
Zinc chloride	1 μM
Iron(III) chloride	55 μM
Thiamine hydrochloride	1 mM
Hydrogen chloride	20 μM
Trisodium citrate	50 μM
MOPS modified buffer	
MOPS	40 mM
Tricine	4 mM
Iron(III) sulfate	0.1 mM
Sodium sulfate	0.276 mM
Calcium chloride	0.5 μM
Magnesium chloride	0.525 mM
Sodium chloride	50 mM
Ammonium molybdate	3 nM
Boric acid	0.4 μM
Cobalt chloride	30 nM
Cupric sulfate	10 nM
Manganese(II) chloride	80 nM
Zinc sulfate	10 nM
Potassium phosphate monobasic	1.32 mM

For both turbidostat and mother machine experiments, chloramphenicol was added at concentrations between 1 and 4.2 μM during translational inhibition experiments. All experiments were performed at 37°C in a climate-controlled environmental room which housed the multiplex turbidostat and all optical components (Darwin Chambers Company, MO). Tables 1 and 2 enumerate experimental conditions and sample sizes for the turbidostat and mother machine experiments, respectively.

### Microscopy configuration.

We performed phase-contrast and fluorescent imaging on a Nikon Ti-E inverted microscope with Perfect Focus (PFS) and an LED transmission light source, controlled by Nikon Elements. For the turbidostat experiments, we used a PFS 2, CoolLED pE-100, 60×, 1.4-numerical-aperture (NA) Ph3 oil immersion objective (Nikon CFI Plan Apo DM Lambda 60× Oil) and an Andor Technology Neo scientific complementary metal oxide semiconductor (sCMOS) camera. For fixed-cell phase-contrast imaging, we used exposure times between 50 and 100 ms and 100% transmission power.

For mother machine experiments, we used a PFS 3, Sutter Instruments TLED, 100× 1.45-NA Ph3 oil immersion objective (Nikon CFI Plan Apo DM Lambda 100× Oil), Photometrics Prime 95B sCMOS camera, and Coherent Obis 488LX laser for epifluorescent illumination. For laser epifluorescent illumination, we inserted a rotating diffuser in the optical train to reduce speckle. We also reduced the camera sensor region of interest to flatten the fluorescent illumination profile. We used a Chroma filter cube with a ZT488rdc dichroic mirror and an ET252/50m emission filter. For live-cell phase-contrast imaging, we used a 30-ms exposure time at 100% transmission power at an interval of 1.5 min. For fluorescent imaging, we used a 25-ms or 50-ms exposure time at 25% power and intervals of 3 min. This weak illumination minimized physiological effects due to phototoxicity effects on the cell and allowed steady-state behavior over many hours.

### Turbidostat cell preparation and sample collection.

We grew all precultures at 32°C or 37°C in a water bath shaker at 260 rpm. Seed cultures were inoculated into 1 to 3 ml LB medium from a single colony from an agar plate, streaked no more than 2 days before use. Cells were grown for several hours and then diluted 1,000-fold into the target media without antibiotics and grown until an optical density at 600 (OD_600_) of 0.1 was reached. If multiple back-dilution rounds were needed to control experimental timing, they were done such that the cells did not enter stationary phase. The culture was then inoculated into each turbidostat vial with or without antibiotics to reach the target OD_600_ of 0.05. Cultures were grown for a minimum of 14 doublings to ensure steady-state conditions upon sample collection. For some conditions, cells adhered to the glass culture vial, evidence of residual biofilm activity that we observed as changes in growth rate over the time course. In these cases, the sample was transferred to a clean glass vial at the end of the experiment for at least 1 additional doubling from which the growth rate was determined.

We collected samples for cell size and cell cycle measurements at OD_600_ of 0.2. Approximately 20 ml of cell culture was immediately put on ice to arrest growth. The culture was then split and pelleted, frozen, or fixed according to the subsequent measurement protocol. A description of our turbidostat design and function has been previously described (3).

### Turbidostat growth rate measurement.

The turbidostat was used to maintain cells growing exponentially between OD_600_ levels of 0.05 and 0.2. In effect, it was run as a batch growth repeater by diluting the culture to an OD_600_ of 0.05 when it reached an OD_600_ of 0.2. An exponential line was fitted to the growth periods between consecutive dilution events. From the exponential line equation *I* = *I*0 · 2*^t^*^/τ^, the growth rate was determined as λ = ln 2/τ, where τ is the doubling time. The turbidostat spectrometers were blanked with the appropriate medium before each experiment.

### Turbidostat cell size measurement.

We fixed cells with a glutaraldehyde and paraformaldehyde mixture and imaged the results within 24 h as previously reported (59), except for the following modifications: 2 μl 25% glutaraldehyde was added to 1 ml 16% paraformaldehyde, and cells were resuspended in 300 μl GTE (50 mM glucose, 25 mM Tris at pH 8.0, 10 mM EDTA at pH 8.0) per sample after phosphate-buffered saline (PBS) washes.

Before imaging, we adjusted cells to an appropriate cell density as needed. Cells were pipetted onto a 2% agarose pad and briefly dried. The agarose pad was then flipped onto a Willco dish (WillCo Wells, Netherlands) and covered with a glass coverslip to reduce evaporation during imaging. A total of 80 to 200 images were generated for each experiment. Sample sizes are presented in Table 1.

We performed fixed-cell image analysis with a custom Python script using the OpenCV library. First, we detected contours using an active Snake’s edge detection algorithm. We then filtered for cell contours using *a priori* knowledge of cell size and shape and manually checked for correctly segmented cells. Width and length were calculated from the long and short axes of the cell segments using a simple threshold analysis of the raw phase-contrast images. All segmented cells for which the width and length fell within 3 standard deviations of the mean for that measurement were kept for further analysis. To calculate cell volume, we assumed the cell was a cylinder with hemispherical ends.

### Turbidostat C period measurement using qPCR.

We estimated the C period using qPCR and marker frequency analysis. Genomic DNA was prepared from each turbidostat sample using a standard phenol chloroform extraction method. We amplified genomic DNA using PowerUp SYBR green master mix (Thermo Fisher Scientific). We used primer pairs targeting chromosomal loci and calculated the C period using the ratio of relative locus copy numbers as discussed previously (3). Primers are listed in Table 6.

**TABLE 6:** qPCR primers

Primer name	Sequence	Location g on genome (*ori* = 0; *ter* = 1)
SJO1152	CGTTGATAGGAACTAGTAGGGA	*ori* forward (right arm)
SJO1153	AGCATTTCGCTCAAGGATG	*ori* reverse (right arm)
SJO1232	GGAATTTCTTTCTCAGGAGAACATTTG	0.2 forward (right arm)
SJO1233	TCTTTATAACGCAGGCATACGG	0.2 reverse (right arm)
SJO1167	CAGTTCGAGCGAAACGATAGA	0.4 forward (right arm)
SJO1168	CGCCACTTTCTCCCTCATAC	0.4 reverse (right arm)
SJO1136	AGAGATGGGTACGATTGTTTG	0.73 forward (right arm)
SJO1137	TTGTCCGCAGCAAGTTC	0.73 reverse (right arm)
SJO1138	TTAACTCGGACATCTTCATCAG	*ter* forward
SJO1139	CAAGGATCAGGAGCAGTTTAT	*ter* reverse
SJO1140	CAGTTCTGCGTTTAGCTGTA	−0.74 forward (left arm)
SJO1141	TTCGGTCATTCTTGTGATAGTT	−0.74 reverse (left arm)
SJO1175	TCAAACACATACTTACTCGGATACA	−0.41 forward (left arm)
SJO1176	CTTGCAGGATTTGAAAGGGAAA	−0.41 reverse (left arm)
SJO1177	CATAACCGGGTACTGAGGAAA	−0.22 forward (left arm)
SJO1178	TCGGATTACGGAAGTTGAAGAG	−0.22 reverse (left arm)
SJO1179	CACTGCCAGCATATTGTTTATCG	*ori* forward (left arm)
SJO1180	GAATGGTTGATCGGTATGGCTA	*ori* reverse (left arm)

### Mother machine cell preparation and image acquisition.

We prepared the cultures for the mother machine experiments using the same method as that previously described for the turbidostat experiments except for the following difference: for translational inhibition experiments, the culture was diluted into the target media with appropriate antibiotics and allowed to grow for several generations before being loaded into the device.

We performed mother machine experiments as previously described (6, 7). We used a custom centrifuge to load cells into the growth channels of the mother machine. The amount of time required to remove cells from the water bath shaker, load them into the growth channels, and infuse fresh 37°C media was between 15 and 30 min. We then imaged cells for many hours under conditions of constant infusion of media via the use of a syringe pump (Harvard Apparatus, MA).

For nutrient shift experiments, two syringe pumps were used in conjunction with a manual Y valve near the device inlet. Cells experienced the change in nutrients in a time interval shorter than the imaging interval (40).

### Mother machine image processing.

Mother machine images were processed with custom Python software (20). The pipeline employs raw images to produce objects which represent a cell and contain all measured parameters. Briefly, the software aligns and crops images into single growth channels (cell traps), segments cells, and links segments in time to construct cell lives and lineages. Segmentation was accomplished with a convolutional neural network of the U-net architecture using manually annotated training data (60). From the constructed cells, we extracted physical parameters in space and time such as size and growth rate. The pipeline can be accessed at https://github.com/junlabucsd/mm3.

After segmentation and lineage creation, the resulting cells were filtered for those with measured parameters (septum position, elongation rate, generation time, birth, division, and added length) within 4 standard deviations of their respective population means. We considered only the cells in the time interval for which the measured parameters and the fluorescent signal were in the steady state. This was normally the interval from 3 to 4 h after imaging began until imaging ceased. For the glycerol-rich growth condition with 3.5 μM chloramphenicol, we excluded cells which divided at the quarter positions, which represented less than 5% of all cells. For all conditions, we further selected a subset of cells which could be followed for at least 4 to 6 consecutive generations. The later filtering step did not affect the parameter distributions but did ensure that cell cycle determinations were possible in light of the presence of overlapping cell cycles. We considered only mother cells during analysis; however, the other cells along the channel had identical elongation rates.

The variability of the physiological parameters is influenced by the imaging frequency. Thus, the CVs in the manuscript represent an upper bound on the true biological CVs. Specifically, the measurements of time were only as precise as the imaging frequency. Measurements of size were similarly limited by how much a cell was able to grow in the imaging interval. For example, we measured division length at the image time point at which we detected division, but the true division event happened somewhere between that time point and the previous time point. We used an imaging frequency of 1.5 min for phase contrast; thus, the worst case (the condition promoting fastest growth) produced 16 images per generation on average. This corresponds to a possible 4% error in measurement of the length at division. This potential error decreased as the imaging frequency increased relative to the generation time or C period. Under most of our conditions, the possible error rate was 1% to 2%. This error source does not apply to measurements of growth rate, width, or septum position.

### Single-cell cycle analysis.

As described in the main text, we used a functional fluorescent DnaN-mGFPmut2 fusion protein. The construct was integrated at the chromosomal locus and expressed under the native promoter. The same genetic configuration was used for E. coli with DnaN-YPet. The gene product was the β-clamp subunit of DNA polymerase III, which is present at high stoichiometry in active replisomes (57).

Cell cycle analysis was performed as described previously (7). Processed fluorescent images were used to determine the cell cycle parameters manually. We first identified replisome foci in the processed fluorescent images using a Laplacian of Gaussian blob detection method. We then constructed cell traces by plotting cell length versus time, with both the fluorescent signal and focus positions projected against the long axis of the cell as demonstrated in Fig. 3. Using an interactive program, we determined the start and end of replication visually based on the positions and numbers of detected foci. For the two conditions promoting fastest growth, i.e., glycerol-rich media with 0 and 1 μM chloramphenicol, the termination time and thus the C period and D period were not determined separately.

### Ensemble cell cycle analysis.

In the ensemble method, we aligned cells by size and plotted the ensemble replication state. On the basis of measurements published by us and others (11), we chose alignment by size rather than cell age. To create the ensemble, we found the average number of foci as a function of cell size across all cells. For the slow-growth case, the number of foci is 1 at short lengths until a transition period, at which point it rises to and plateaus at 2. We take the initiation length to be the length at which the focus count rate of change is the highest, using a differentiation step corresponding to 0.2 μm. By inferring the average number of overlapping cell cycles (*n*_oc_) from the traces, we can calculate C + D as follows: C + D = [*n*_oc_ + log_2_ (*S_d_*/*S_i_*)] · τ.

### Data availability.

Single-cell data from the steady-state mother machine experiments are provided in Data Set S1. All other data from the study are available upon request.

10.1128/mBio.02205-19.10 DATA SET S1 Single-cell data for steady-state mother machine experiments. Download Data Set S1, XLSX file, 2.5 MB. Copyright © 2019 Sauls et al. 2019 Sauls et al. This content is distributed under the terms of the Creative Commons Attribution 4.0 International license.
