# Supplementary figures and images for: Control of Bacillus subtilis Replication Initiation during Physiological Transitions and Perturbations
Source: mBio. 2019 Dec 17;10(6):e02205-19. doi: 10.1128/mBio.02205-19 (PMC6918070; doi:10.1128/mBio.02205-19)

# Mother machine steady-state measurements from all conditions

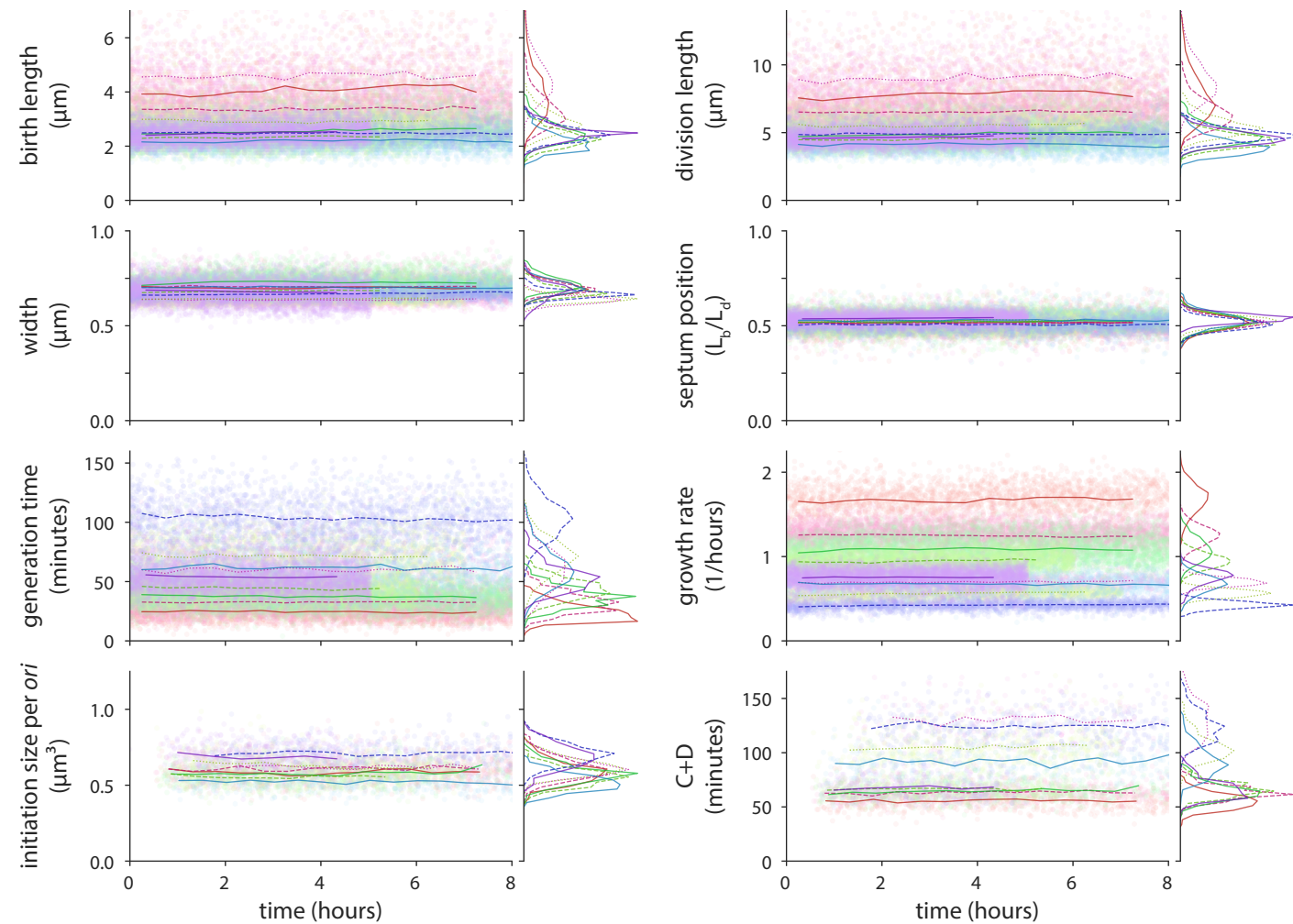

Supplement: FIG S1 [file mBio.02205-19-sf001.pdf]

A Cell length versus growth rate

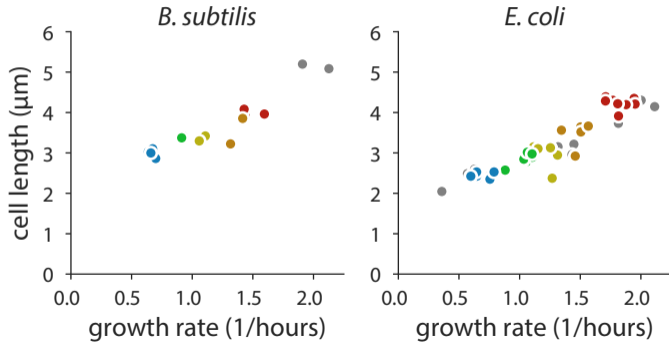

B Cell width versus growth rate

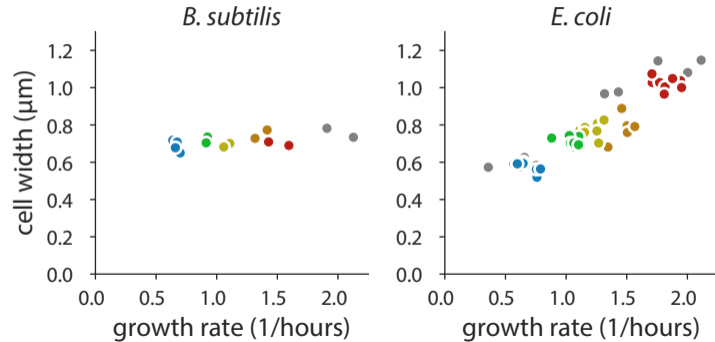

Supplement: FIG S2 [file mBio.02205-19-sf002.pdf]

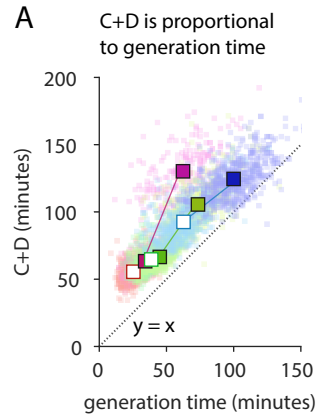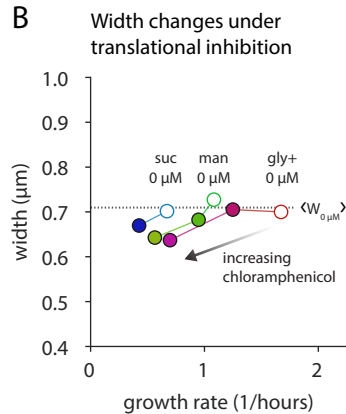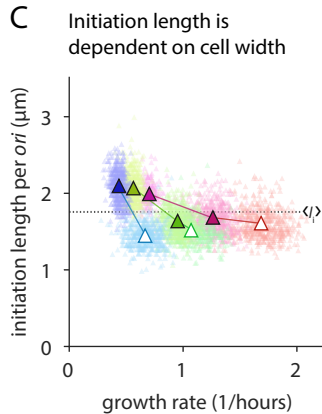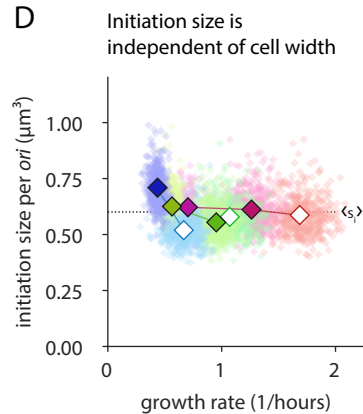

Supplement: FIG S5 [file mBio.02205-19-sf005.pdf]

Normalized cross correlations of  
physiological parameters for all conditions

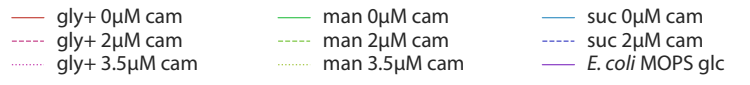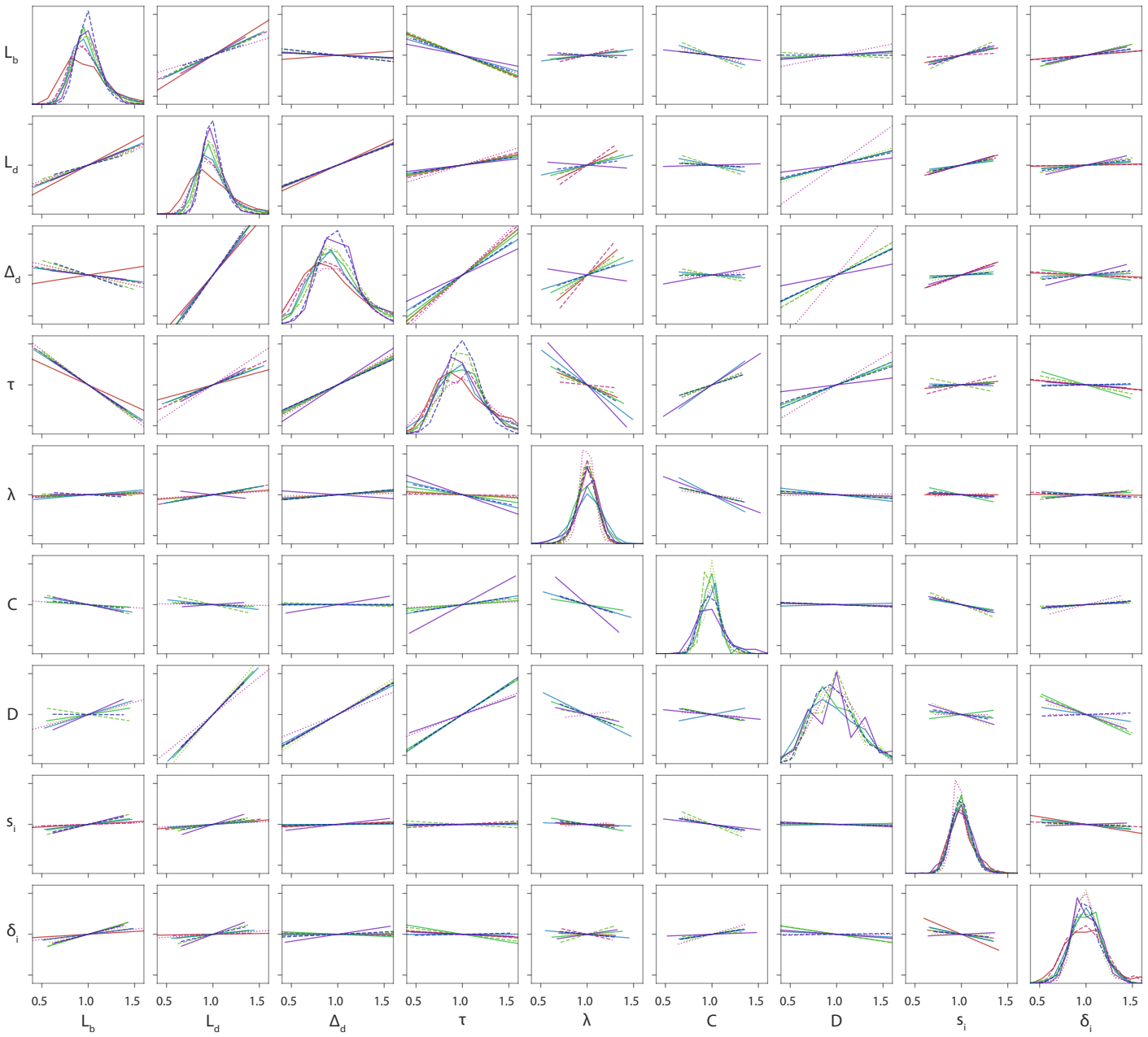

Supplement: FIG S9 [file mBio.02205-19-sf009.pdf]

# A Single-cell behavior during nutrient shift-up and shift-down

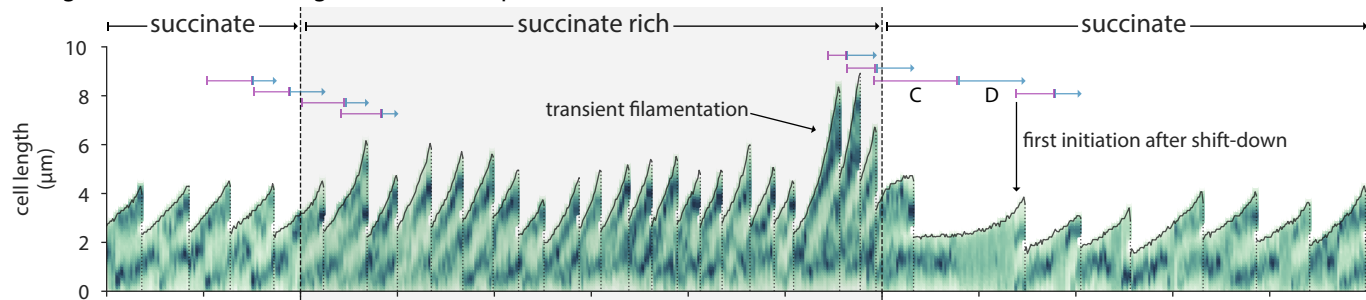

# B Population average

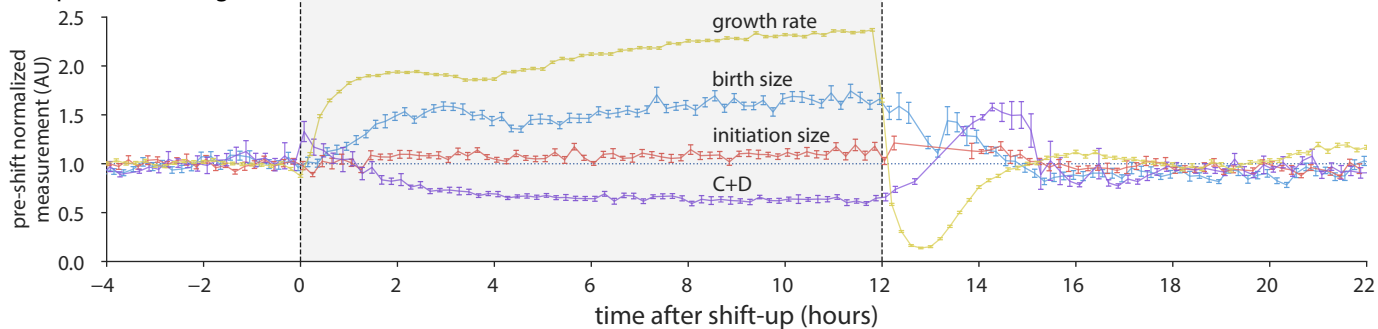

Supplement: FIG S6 [file mBio.02205-19-sf006.pdf]
