# Supplementary material for: Control of Bacillus subtilis Replication Initiation during Physiological Transitions and Perturbations
Source: mBio. 2019 Dec 17;10(6):e02205-19. doi: 10.1128/mBio.02205-19 (PMC6918070; doi:10.1128/mBio.02205-19)
Supplement: FIG S3 [file mBio.02205-19-sf003.pdf]

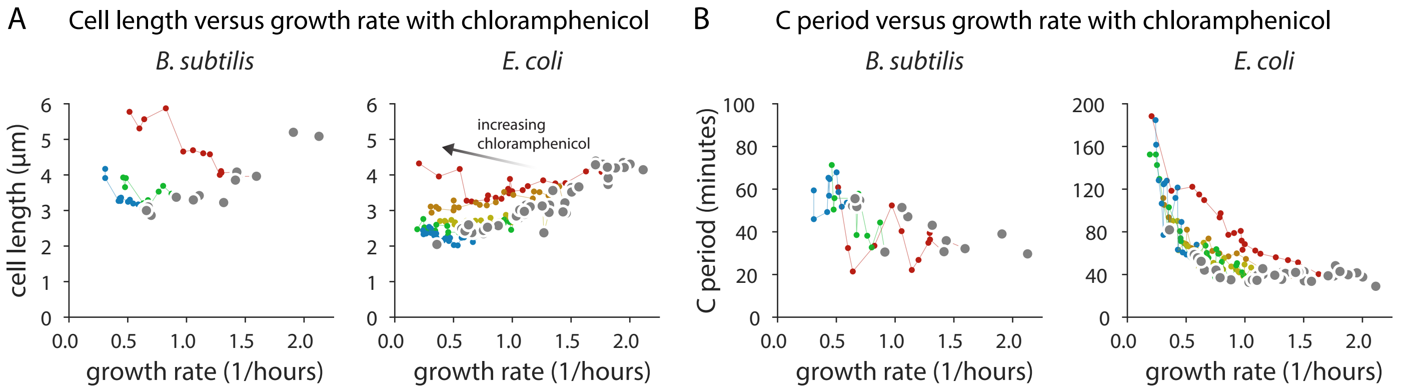
A
Cell length versus growth rate with chloramphenicol
B. subtilis
E. coli
cell length (µm)
growth rate (1/hours)
increasing
chloramphenicol
0
1
2
3
4
5
6
0.0
0.5
1.0
1.5
2.0
B
C period versus growth rate with chloramphenicol
B. subtilis
E. coli
C period (minutes)
0
20
40
60
80
100
0
40
80
120
160
200
