# Supplementary material for: Control of Bacillus subtilis Replication Initiation during Physiological Transitions and Perturbations
Source: mBio. 2019 Dec 17;10(6):e02205-19. doi: 10.1128/mBio.02205-19 (PMC6918070; doi:10.1128/mBio.02205-19)
Supplement: FIG S4 [file mBio.02205-19-sf004.pdf]

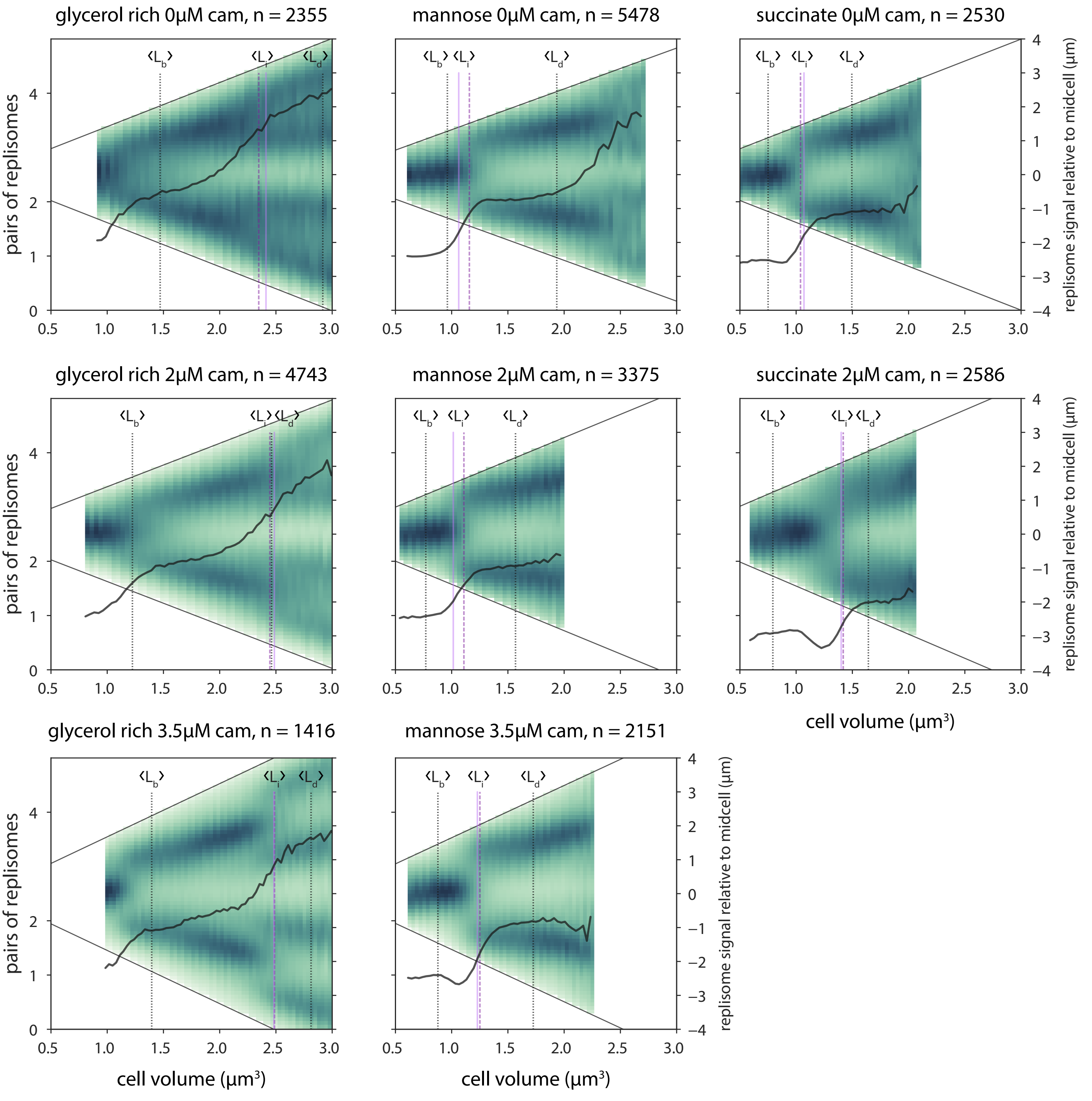

glycerol rich 0μM cam, n = 2355
mannose 0μM cam, n = 5478
succinate 0μM cam, n = 2530
glycerol rich 2μM cam, n = 4743
mannose 2μM cam, n = 3375
succinate 2μM cam, n = 2586
glycerol rich 3.5μM cam, n = 1416
mannose 3.5μM cam, n = 2151
⟨Lb⟩
⟨Li⟩
⟨Ld⟩
pairs of replisomes
replisome signal relative to midcell (μm)
cell volume (μm³)
