# Supplementary material for: Control of Bacillus subtilis Replication Initiation during Physiological Transitions and Perturbations
Source: mBio. 2019 Dec 17;10(6):e02205-19. doi: 10.1128/mBio.02205-19 (PMC6918070; doi:10.1128/mBio.02205-19)
Supplement: FIG S8 [file mBio.02205-19-sf008.pdf]

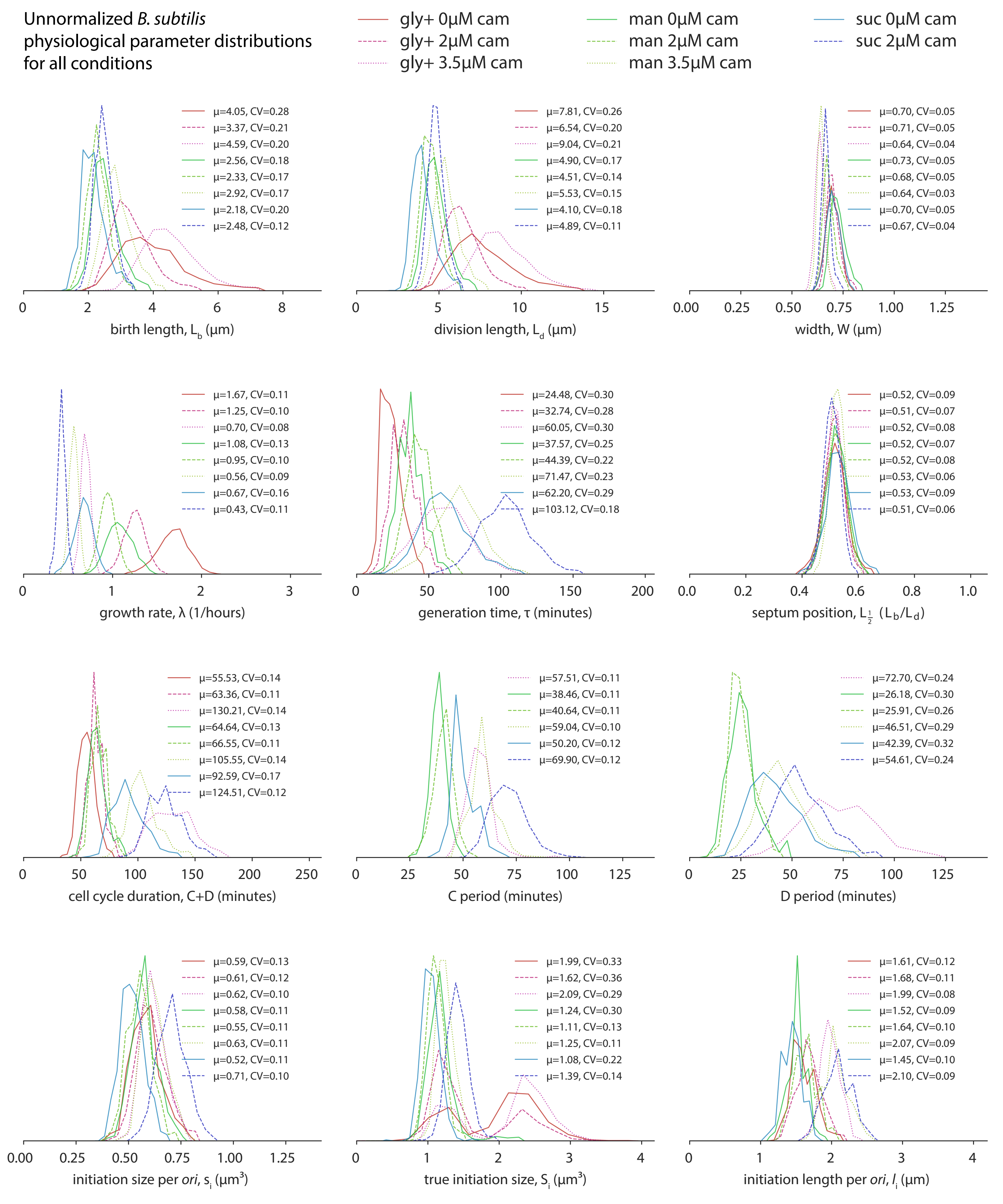
Unnormalized B. subtilis physiological parameter distributions for all conditions
gly+ 0μM cam
gly+ 2μM cam
gly+ 3.5μM cam
man 0μM cam
man 2μM cam
man 3.5μM cam
suc 0μM cam
suc 2μM cam
μ=4.05, CV=0.28
μ=3.37, CV=0.21
μ=4.59, CV=0.20
μ=2.56, CV=0.18
μ=2.33, CV=0.17
μ=2.92, CV=0.17
μ=2.18, CV=0.20
μ=2.48, CV=0.12
0 2 4 6 8
birth length, L_b (μm)
μ=7.81, CV=0.26
μ=6.54, CV=0.20
μ=9.04, CV=0.21
μ=4.90, CV=0.17
μ=4.51, CV=0.14
μ=5.53, CV=0.15
μ=4.10, CV=0.18
μ=4.89, CV=0.11
0 5 10 15
division length, L_d (μm)
μ=0.70, CV=0.05
μ=0.71, CV=0.05
μ=0.64, CV=0.04
μ=0.73, CV=0.05
μ=0.68, CV=0.05
μ=0.64, CV=0.03
μ=0.70, CV=0.05
μ=0.67, CV=0.04
0.00 0.25 0.50 0.75 1.00 1.25
width, W (μm)
μ=1.67, CV=0.11
μ=1.25, CV=0.10
μ=0.70, CV=0.08
μ=1.08, CV=0.13
μ=0.95, CV=0.10
μ=0.56, CV=0.09
μ=0.67, CV=0.16
μ=0.43, CV=0.11
0 1 2 3
growth rate, λ (1/hours)
μ=24.48, CV=0.30
μ=32.74, CV=0.28
μ=60.05, CV=0.30
μ=37.57, CV=0.25
μ=44.39, CV=0.22
μ=71.47, CV=0.23
μ=62.20, CV=0.29
μ=103.12, CV=0.18
0 50 100 150 200
generation time, τ (minutes)
μ=0.52, CV=0.09
μ=0.51, CV=0.07
μ=0.52, CV=0.08
μ=0.52, CV=0.07
μ=0.52, CV=0.08
μ=0.53, CV=0.06
μ=0.53, CV=0.09
μ=0.51, CV=0.06
0.0 0.2 0.4 0.6 0.8 1.0
septum position, L_½ (L_b/L_d)
μ=55.53, CV=0.14
μ=63.36, CV=0.11
μ=130.21, CV=0.14
μ=64.64, CV=0.13
μ=66.55, CV=0.11
μ=105.55, CV=0.14
μ=92.59, CV=0.17
μ=124.51, CV=0.12
0 50 100 150 200 250
cell cycle duration, C+D (minutes)
μ=57.51, CV=0.11
μ=38.46, CV=0.11
μ=40.64, CV=0.11
μ=59.04, CV=0.10
μ=50.20, CV=0.12
μ=69.90, CV=0.12
0 25 50 75 100 125
C period (minutes)
μ=72.70, CV=0.24
μ=26.18, CV=0.30
μ=25.91, CV=0.26
μ=46.51, CV=0.29
μ=42.39, CV=0.32
μ=54.61, CV=0.24
0 25 50 75 100 125
D period (minutes)
μ=0.59, CV=0.13
μ=0.61, CV=0.12
μ=0.62, CV=0.10
μ=0.58, CV=0.11
μ=0.55, CV=0.11
μ=0.63, CV=0.11
μ=0.52, CV=0.11
μ=0.71, CV=0.10
0.00 0.25 0.50 0.75 1.00 1.25
initiation size per ori, s_i (μm³)
μ=1.99, CV=0.33
μ=1.62, CV=0.36
μ=2.09, CV=0.29
μ=1.24, CV=0.30
μ=1.11, CV=0.13
μ=1.25, CV=0.11
μ=1.08, CV=0.22
μ=1.39, CV=0.14
0 1 2 3 4
true initiation size, S_i (μm³)
μ=1.61, CV=0.12
μ=1.68, CV=0.11
μ=1.99, CV=0.08
μ=1.52, CV=0.09
μ=1.64, CV=0.10
μ=2.07, CV=0.09
μ=1.45, CV=0.10
μ=2.10, CV=0.09
0 1 2 3 4
initiation length per ori, l_i (μm)
