# Supplementary material for: Control of Bacillus subtilis Replication Initiation during Physiological Transitions and Perturbations
Source: mBio. 2019 Dec 17;10(6):e02205-19. doi: 10.1128/mBio.02205-19 (PMC6918070; doi:10.1128/mBio.02205-19)
Supplement: FIG S7 [file mBio.02205-19-sf007.pdf]

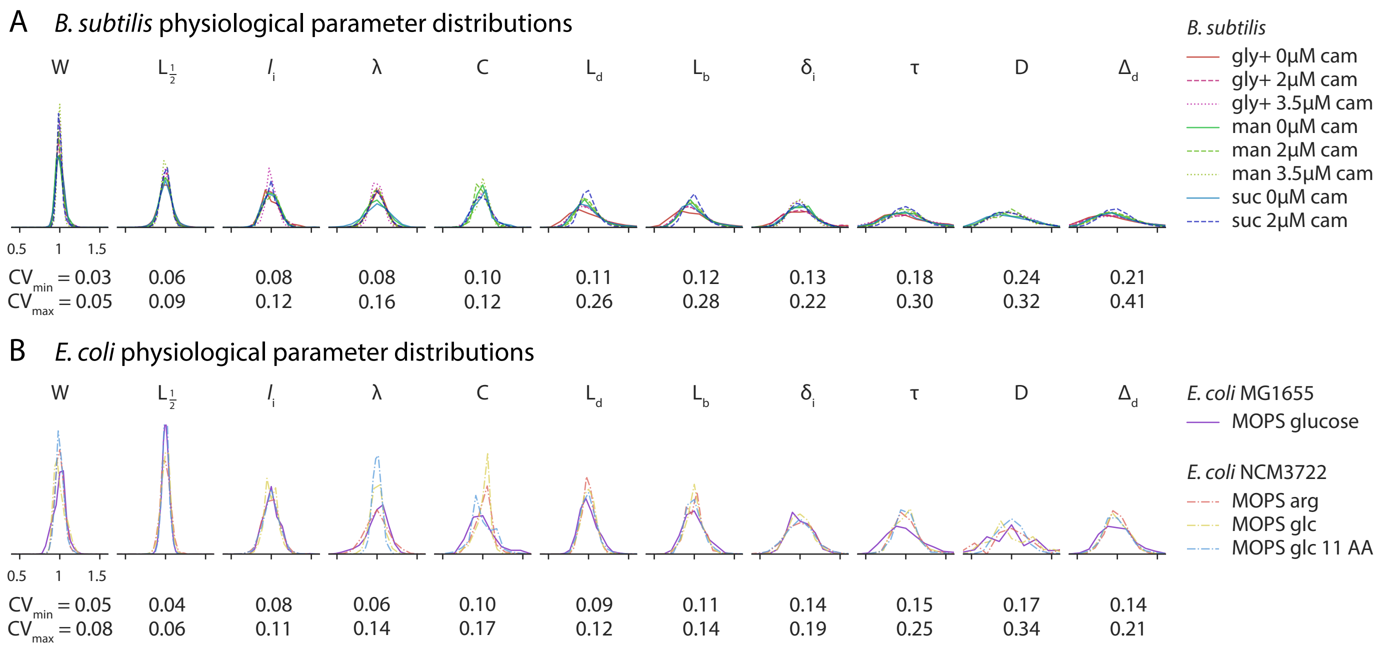

A
B. subtilis physiological parameter distributions
W
L1/2
li
λ
C
Ld
Lb
δi
τ
D
Δd
0.5
1
1.5
CVmin = 0.03
0.06
0.08
0.08
0.10
0.11
0.12
0.13
0.18
0.24
0.21
CVmax = 0.05
0.09
0.12
0.16
0.12
0.26
0.28
0.22
0.30
0.32
0.41
B. subtilis
gly+ 0μM cam
gly+ 2μM cam
gly+ 3.5μM cam
man 0μM cam
man 2μM cam
man 3.5μM cam
suc 0μM cam
suc 2μM cam
B
E. coli physiological parameter distributions
W
L1/2
li
λ
C
Ld
Lb
δi
τ
D
Δd
0.5
1
1.5
CVmin = 0.05
0.04
0.08
0.06
0.10
0.09
0.11
0.14
0.15
0.17
0.14
CVmax = 0.08
0.06
0.11
0.14
0.17
0.12
0.14
0.19
0.25
0.34
0.21
E. coli MG1655
MOPS glucose
E. coli NCM3722
MOPS arg
MOPS glc
MOPS glc 11 AA
